# Editorial: Targeting ion homeostasis for cancer therapy: mechanisms and immunomodulatory effects

**DOI:** 10.3389/fphar.2024.1418700

**Published:** 2024-05-08

**Authors:** Xuemei Zeng, Zhenglin Li, Zhiyao Yi, Shuangqian Yan

**Affiliations:** ^1^ Key Laboratory of Microbial Pathogenesis and Interventions of Fujian Province University, Biomedical Research Center of South China, College of Life Sciences, Fujian Normal University, Fuzhou, China; ^2^ Oujiang Laboratory (Zhejiang Lab for Regenerative Medicine, Vision and Brain Health), School of Pharmaceutical Sciences, Wenzhou Medical University, Wenzhou, Zhejiang, China; ^3^ Department of Chemistry, The N1 Institute for Health, National University of Singapore, Singapore, Singapore; ^4^ The Straits Institute of Flexible Electronics (SIFE, Future Technologies), Straits Laboratory of Flexible Electronics (SLoFE) Fujian Normal University, Fuzhou, Fujian, China

**Keywords:** ion homeostasis, ferroptosis, cuproptosis, immunotherapy, STING

Ions play crucial roles in cellular activities and overall health. These ions encompass both metals (such as Ca^2+^, Zn^2+^, Mn^2+^, Fe^2+^/Fe^3+^, K^+^, Na^+^, and Mg^2+^) and non-metals (such as Cl^−^, CO_3_^2−^, PO_4_^3−^, and NO_3_^−^), contributing not only to the osmotic pressure of bodily fluids but also governing significant functions in organismal development and disease occurrence. Consequently, maintaining ion homeostasis is pivotal for disease treatment, particularly in tumor therapy. For example, calcium, zinc, and sodium ions have been associated with cellular apoptosis. Various ions with diverse concentrations can induce cell death by generating reactive oxygen species through Fenton or Fenton-like reactions. Iron-mediated ferroptosis has emerged as a promising approach for tumor treatment, which can be initiated not only by an excess of iron ions but also by zinc, manganese, and copper. Moreover, recent findings have highlighted that excess copper can trigger cellular cuproptosis, representing a newly defined form of regulated cell death. These ions also interact with biological metabolisms and immune systems. Thus, exploring the relationships between ions and biological pathways not only enhances our understanding of their biological functions but also suggests potential strategies for disease control.

This Research Topic comprises six articles, including reviews and original research, shedding new light on ion-related cell death, glutamine metabolism, and meta-analysis of immune checkpoint blockers. Furthermore, it offers insights into nanoparticles for disease control and the impact of ions on T cell stemness in cancer immunotherapy. Below is a succinct overview of the articles featured in this Research Topic:

Liu et al. provide a review highlighting the significance of T cell stemness in cancer immunotherapy. They discuss how the tumor microenvironment, characterized by hypoxia and heightened immunosuppression, creates favorable conditions for tumor cells while leading to T cell exhaustion. The article begins by examining the status and stemness of T cells within the tumor microenvironment. It then delves into the impact of tumoral factors such as K^+^, H^+^, and lactate on T cell function and stemness, elucidating their crucial role in modulating antitumor immune responses.

Fan et al. present a review that emphasizes the role of glutamine metabolism in tumor progression while highlighting the potential targets for tumor therapy. They outline the various pathways of glutamine metabolism in cancer cells and analyze how these pathways contribute to tumor development. Additionally, the authors examine the role of glutamine metabolism in immune cells, including macrophages and lymphocytes. Furthermore, they provide an overview of drugs targeting glutamine metabolism for cancer therapy, such as glutaminase inhibitors, glutamine uptake inhibitors, glutamate dehydrogenase inhibitors, and aminotransferase inhibitors. Finally, they discuss the challenges associated with targeting glutamine metabolism and offer future perspectives for its modulation in cancer treatment.

In another study, Han et al. conducted a meta-analysis to assess the efficacy and safety of programmed cell death protein 1 (PD-1) and programmed death-ligand 1 (PD-L1) inhibitors in endometrial cancers. Their findings revealed that the total objective response rate (ORR) among patients with advanced or recurrent endometrial cancer and mismatch repair-deficient (dMMR) status was 51.9%, whereas it was only 16.1% for patients with mismatch repair-proficient (pMMR) status. These results indicate that patients with dMMR may derive greater benefits from PD-1/PD-L1 inhibitors compared to those with pMMR, offering valuable insights for the selection of immunomodulators in endometrial cancer therapy in clinical practice.

Sobhani-Nasab et al. present a comprehensive review focusing on nanoparticles for disease treatment across various domains, including bacterial infection, tuberculosis, infectious diseases, dentistry, cancer therapy, neurodegenerative diseases, and tissue repair and regeneration. These nanoparticles not only function as drug delivery systems but also possess intrinsic biological or specific physicochemical properties essential for disease therapy. Additionally, the authors delve into the potential toxicity of nanoparticles in future applications.

Feng et al. conducted an analysis of ten genes (GYS1, NDUFS1, OXSM, LRPPRC, NDUFA11, NUBPL, NCKAP1, RPN1, SLC3A2, and SLC7A11) associated with disulfide stress-induced cell death, known as disulfidoptosis, in patients with kidney renal clear cell carcinoma using a database. They elucidated the expression profiles of disulfidoptosis-related genes and their chromosomal mappings, highlighting their potential as prognostic markers and offering potential targets for therapy in kidney renal clear cell carcinoma.

Excess copper ions within cells can directly bind to lipoylated proteins within the tricarboxylic acid cycle, leading to proteotoxic stress through copper-mediated lipoylated protein aggregation and subsequent loss of iron-sulfur cluster proteins such as ferredoxin 1 (PDX1). This novel form of copper-dependent cell death, termed “cuproptosis,” was identified in 2022. However, a more comprehensive understanding of the molecular mechanisms underlying cuproptosis is needed. In this Research Topic, Lv et al. discovered a positive correlation between yes-associated protein 1 (YAP1), a downstream oncogene of the Hippo pathway, and PDX1 in the skin cutaneous melanoma cancer line, A2058. Furthermore, they observed a correlation between YAP1 and infiltration of macrophages and regulatory T cells. This study unveils the association of YAP1 with cuproptosis and immune infiltration.

In conclusion, this Research Topic has shed light on the critical roles of ions in cellular activities and health maintenance, emphasizing their significance in disease treatment, particularly in tumor therapy. By exploring the relationships between ions and biological pathways, we not only enhance our understanding of their biological functions but also identify potential strategies for disease control. Furthermore, the Research Topic addresses various aspects of ion-related cell death, glutamine metabolism, immune checkpoint blockers, nanoparticles for disease control, and T cell stemness in cancer immunotherapy. Each article provides valuable insights into the complex mechanisms underlying disease processes and offers potential avenues for therapeutic intervention. These studies underscore the importance of multidisciplinary approaches in advancing our understanding of disease mechanisms and developing novel therapeutic strategies. Moving forward, continued research in these areas will be instrumental in translating scientific discoveries into clinical applications, ultimately improving patient outcomes and advancing the field of medical science.

